# The Relationship between Research and Casework in Forensic Entomology

**DOI:** 10.3390/insects12020174

**Published:** 2021-02-17

**Authors:** Martin J. R. Hall

**Affiliations:** Department of Life Sciences, Natural History Museum, London SW7 5BD, UK; m.hall@nhm.ac.uk

**Keywords:** casework, court, criminal justice systems, expert witness, forensic entomology, insect evidence, research

## Abstract

**Simple Summary:**

Forensic entomology concerns the use of insects as evidence in legal investigations. Many sorts of investigation can benefit from an interpretation of insects associated with the crime scene, but insect evidence is most frequently used in investigations of death. The interpretation of insect evidence in casework is guided by the data supplied through research. Such data are essential to improve the casework interpretation of insect evidence, thereby improving the robustness of the legal systems in which it operates. This paper explores the mutually beneficial relationship between research and casework in forensic entomology, contrasting the different challenges that each presents and giving examples of how each can support the other in delivering results of real societal benefit. It is written from the perspective of the Criminal Justice System of England and Wales, but many of the points raised are relevant to legal systems worldwide.

**Abstract:**

Research is a vital component of all forensic sciences and is often stimulated by casework, which identifies gaps in our knowledge. In such a niche area of forensic science as entomology there should be a close and mutually beneficial relationship between research and casework: to some extent there is a continuum between the two and many forensic entomologists are involved in both to a greater or lesser degree. However, research and casework involve quite differing challenges, from the replicated, highly controlled, sometimes esoteric aspects of research to the very individual, sometimes chaotic and disruptive, but highly applied aspects of casework. Ideally casework will include the full involvement of a forensic entomologist, who will collect the insect and climate evidence at the scene and produce a robust expert witness statement based on a full analysis of this data. Unfortunately, it can also include situations where samples, if collected at all, are poorly preserved, not representative of the full cadaver fauna available and presented to the entomologist months or years after the event, without local temperature data. While research is recognised through publications and their citation indices, casework and its associated expert witness statements often receive no credit in an academic workplace, although they do have a positive societal impact and many other benefits of teaching and public engagement value. This manuscript examines the relationship between research and casework from a UK perspective, to raise awareness of the need to create an environment that values the contribution of both, for future generations to flourish in both areas.

## 1. Introduction

Before the 1980s, research publications in the field of forensic entomology were relatively infrequent [1] (p. 418), and therefore the research support to investigations of insect evidence during casework was limited. The first case in which forensic entomology evidence was used in the UK was the “Ruxton” case of 1935 [2]. Analysis of the insect evidence in this case was based on records of insect development that were unpublished at the time, nevertheless it was important as corroboration of other evidence even if not used in court [3,4].

Publication of Ken Smith’s Manual of Forensic Entomology in 1986 [5] was a major catalyst to research, bringing together the widely scattered forensic entomology literature available at that time and combining it with relevant taxonomic and ecological literature recording the biology of carrion fauna. I believe that it is no coincidence that its publication was in the decade that saw the start of an almost exponential rise in scientific publications in the field of forensic entomology [1] (p. 418), which continues to increase year-on-year [6]. A substantial portion of Smith’s book was devoted to insect identification, highlighting the critical importance of assigning the correct name to insect evidence at the start of any casework. This landmark book clearly demonstrated the potential value of insect evidence in criminal investigations, summarizing 19 case histories and suggesting areas for future research of value to forensic entomology casework, thereby being of importance in highlighting the relationship between research and casework.

In 2009, Magni et al. [7] published results of a survey of forensic entomologists worldwide. Of some 300 individuals contacted, 70 responded and by far the largest group (60%) were employed in universities. However, 67% of all respondents considered forensic entomology to be only their secondary occupation. Some 79% of respondents published in the area of forensic entomology or taxonomy while a similar proportion, 74%, undertook casework. Nevertheless, only 25% of the caseworkers had worked on >100 cases, most (64%) had worked on <50 cases and half of those on <10 cases. In my opinion, it seems likely that there are many forensic entomologists who spend far more of their time on research (and teaching) than on casework, in part because of the difficulty of gaining casework experience due: (1) to the relatively low number of cases in which it is used; and (2) the generally pragmatic nature of those who require a forensic entomology input to their investigations, minimizing risk and/or honouring service contracts by selecting those they have previously used with success.

The following comparison of research and casework in forensic entomology is very much a personal perspective developed from working within a UK context. The policing and legal systems of the UK are complex and differ between those of Northern Ireland, Scotland and England and Wales—the majority of my experience is within the Criminal Justice System of England and Wales (abbreviated here as CJS-EW). I look at some of the challenges of working in casework within an academic or other scientific institute background, e.g., universities and museums, which Magni et al.’s [7] survey shows to be the dominant workplace for forensic entomologists.

## 2. Research and Casework—Similarities and Differences

Table 1 summarises some of the differences between undertaking research and undertaking casework in forensic entomology, indeed in virtually all branches of forensic science. In addition to the likely requirement for caseworkers of a research degree, such as a PhD, and subsequently acquired practical experience of the subject that qualifies them to provide an expert opinion on specific, insect related aspects of a criminal investigation, it is of great benefit to the forensic entomologist to acquire training in expert witness skills: if only to make them aware of the potentially stressful and intimidating environment of a court room [8], to help in writing a witness statement and to prepare for the likely style of questioning.

Most practicing forensic entomologists can expect to appear in court at some time to be questioned on the opinions and conclusions in their expert witness statements. Of course, court rooms and the legal systems practiced within them vary enormously from country to country. My background is in the adversarial system practiced in the UK and, during my career, I appeared in court in just 12.3% (18/146) of the cases for which I submitted one or more expert witness statements. In some cases that did not result in a court appearance the entomology evidence may either have been submitted, but was not contested, or it may not have had evidential value. Without the training I received from an expert witness training provider, which had been assessed, quality assured and certified by a university school of law [9], I would have been much less comfortable in the court room. In addition to courses, there are a number of published articles and numerous books, e.g., [10,11], to help prepare an expert witness for court, in addition to the online aids available from the judiciaries of England and Wales [12,13] and Scotland [14] and from the office of the Forensic Science Regulator [15,16]. There are also some invaluable aids written specifically for forensic entomologists [17,18]. An interesting and personal perspective on the challenges of presenting forensic entomology evidence in court is given in an opinion paper by Disney [19], discussed by reference to a number of specific cases, which together illustrate the wide variety of ways in which insect evidence can be used.

Major differences between research and casework appear in the actual nature of the study. All research relies on adequate replication [20]. However, each case is a one-off, unique scenario—even apparently similar cases usually vary spatially and temporally. This can present real challenges in interpretation. Sometimes valuable information towards an understanding of the biology of a case can be gathered by simulation of important aspects of the original case, e.g., use of a buried pig to simulate the decomposition of a victim in a shallow grave [21]. However, such simulation is often not possible due to time constraints imposed by the investigation or difficulties in working at the crime scene, especially if indoors, e.g., due to limited access.

While, by and large, a research study can be conducted within a planned schedule to self-managed deadlines, casework is often highly disruptive to the timetables of research and teaching that most forensic entomologists also undertake and it runs to strict deadlines, imposed both by the needs of the investigation and of any subsequent trial. Research is much more amenable to control of its timetable than is casework.

Other than products such as patents, the final products from most research work are peer-reviewed publications in the scientific literature. For these the researcher receives academic credit based on some form of citation indicator [22]. To my knowledge and experience, no academic credit is usually given for the expert witness statements which are the product of a forensic investigation. However, many factors compensate for the lack of standard academic credit and the caseworker can take credit from the knowledge that their evidence has benefited more than science, it has made a positive contribution to society, contributing to the criminal justice system generally as well as to specific individuals, such as relatives of victims who, from personal experience, can benefit from the closure given through insect evidence [23]. Feedback is sometimes received by caseworkers from someone in the judicial system that also demonstrates the societal value of the casework. Three examples of feedback that I have received are given below and illustrate the value of forensic entomology, not only in the court room but earlier in the investigation, for example as a guide to what time period for the investigators to focus on, leading to savings in increasingly limited resources:

- “The opinions you expressed in your statement went a considerable way to support our hypothesis, based on a number of other known facts. Your statement was accepted in evidence by the defence. I was very pleased with the accuracy of the opinions you expressed, which I am sure went someway to causing the defendant to change his account. Had he not changed his account at the eleventh hour we would have relied considerably upon your evidence to convince the jury (of our hypothesis). I am therefore satisfied that the evidence you provided was useful to the case and represented good value for money.”
- “…the investigation team were very happy to be told that death occurred on the day [the victim] went missing as this hugely reduced the amount of CCTV they had to view.” (NB: investigators still had to view >11,000 h of CCTV in this case.).
- “Your statement was crucial in securing guilty pleas. The two accused were pleading not guilty up until the moment of the trial beginning. The trial, had it run, would have lasted a week or so at great expense, so your statement led to those guilty pleas. So, value for money it was worth it.”

In addition to the knowledge that casework is of societal value, engagement in casework provides researchers with practical experience that they can share with students, adding value to their teaching, it provides ideas for projects (e.g., see Section 3 below), it can provide income for the institute and it provides experience for use in public engagement activities.

Writing an expert witness statement can be as challenging as writing a scientific paper, if not more so. While each witness statement is usually founded on a smaller scientific study than a research paper, the implications for the conclusions and, therefore, the consequences of errors, are profound. For forensic entomology cases investigating neglect or death, each statement usually involves a factual account of when and where specimens were collected, how they were processed, how they were identified and how they were aged based on estimated temperatures for the period of development. However, it is not just about facts, but about how these facts are then interpreted based on experience to assist the court. This summation of facts and opinion results in an estimation of the time that the earliest female insect laid her eggs/larvae on the body of the victim, living or dead. In an investigation of death, this equates to the minimum post-mortem interval, minPMI [24,25]. The witness statement can also include a testing of any hypotheses proposed by either prosecution (*H*_p_) or defence (*H*_d_) [26,27,28].

Of course, with some cases it is possible to prepare not only an expert witness statement but also a scientific publication, with details of cases suitably anonymized [29,30,31]. Case reports are widely accepted in medical science, with several journals dedicated to their publication, e.g., *BMJ Case Reports*, *Oxford Medical Case Reports* and *Journal of Medical Case Reports*. Such publications are also of value in forensic science and can be of enormous benefit in describing unusual cases (e.g., [32,33]), providing a comparison of similar cases (e.g., [34,35,36]), identifying local fauna (e.g., [37,38]), and in discussing the application and validation of methods (e.g., [39,40,41,42,43]), especially if there is an independent and accurate verification of time of death to compare with the entomology estimates.

With forensic scientists being under greater scrutiny now than ever before, there is a great deal of responsibility and legal obligations on any expert witness, as detailed in a document produced by the Forensic Science Regulator regarding the CJS-EW [44]. I believe that the pressures on an expert witness have always been there, but they have increased over the last four decades with, for example, greater scrutiny, greater accreditation, shorter time scales and tighter budgets. From the first statement I produced in 1992, the only way that anyone might know that I had any knowledge of entomology was the fact that my address included “Department of Entomology at the Natural History Museum, London”. In contrast, in addition to a detailed statement of my qualifications and experience, my final statement produced in 2019 included a one page declaration with more than 20 bullet points based on guidance from the Criminal Practice Directions [45] (19B, pp. 35–37), essentially declaring awareness of my duties as an expert witness, including that “I am likely to be the subject of public adverse criticism by the judge if the Court concludes that I have not taken reasonable care in trying to meet the standards set out above”. I think this declaration is entirely fair and reasonable but, in its clear and unambiguous statement, it is still a stark reminder of the onus on an expert witness. As mentioned above, the pressure on expert witnesses has always been there, evidenced by the autobiography of Professor Keith Simpson [46] in which he says, “My insistence on the timing of death had become pretty well known, to the police, the Director of Public Prosecutions, the lawyers—and the Press, who would have scented a public disgrace for me if I’d been wrong.” [46] (p. 310). However, in this case, the so-called “Lydney murder”, Professor Simpson had opened himself up to unnecessary pressure by declaring a period of death, rather than a minPMI, at the scene, before he had a chance to confirm the larval identification and take into account the ambient climate conditions [46] (pp. 300–310). His estimate was not unreasonable, but I would not recommend making such an unequivocal statement at a crime scene without caveats.

**Figure 1:**
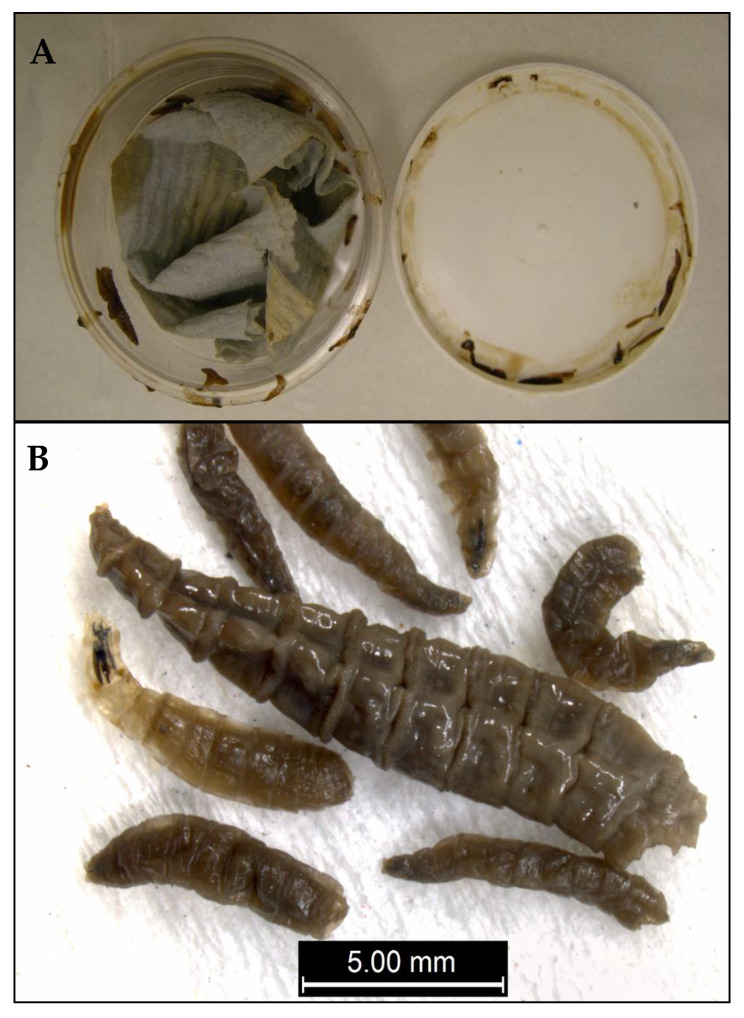
Example of forensic exhibit containing larvae of *Calliphora vomitoria* (Diptera: Calliphoridae) collected alive, but then sealed inside an airtight plastic pot (**A**) inside a plastic evidence bag so that all had died (**B**) by the time of their arrival at the laboratory.

**Figure 2:**
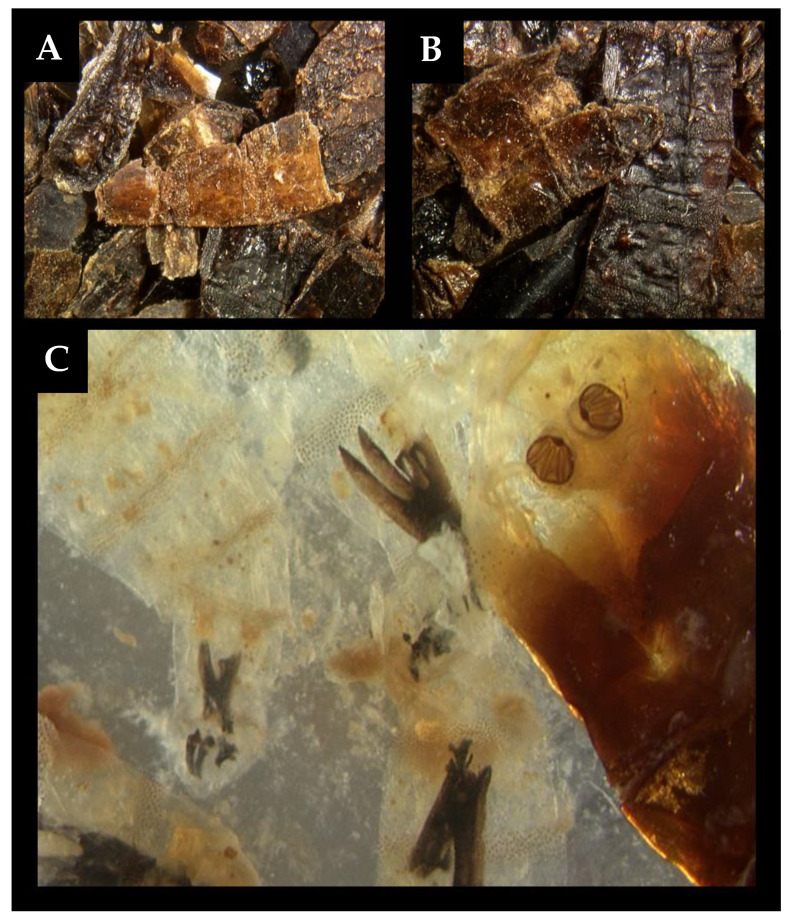
Example of suboptimal maintenance of insect evidence, stored without preservative for 3.5 years in a sealed glass jar in a fridge. (**A**,**B**) dried flattened fragments of fly larvae, resembling flakes of rusted metal; (**C**) oldest specimens revealed to be at 2nd–3rd instar moult and early 3rd instars of *Calliphora vomitoria* (Diptera: Calliphoridae) following immersion in 10% aqueous potassium hydroxide (KOH) [5].

The lack of control of many factors in casework, mentioned above, can make it very difficult to produce a robust expert witness statement. In an ideal world, a forensic entomologist would visit the scene and/or the post-mortem to collect the insect evidence. My own experience is of visiting the scene in just 18.5% (27/146) of the cases for which I produced a statement. Not visiting a scene does not mean that it is impossible to produce a robust statement, but the confidence intervals might have to be widened and more caveats introduced. Additionally, the forensic entomologist can give trained crime scene personnel timely additional advice and instruction by mobile telephone as they collect insect evidence at the scene, and the entomologist can visit the scene virtually when they later view crime scene photographs and videos, including those produced by 360° imaging techniques [47,48]. However, non-attendance at the scene by an entomologist can introduce the potential for complications, illustrated in Table 2: examples of those I have experienced include:

- Provision of just six larvae for analysis when many thousands are evident on scene photographs.
- Puparia (apparent on scene photographs) were overlooked in collections because they were not on the body and did not move, and so were just not considered part of the insect evidence, although they were most likely the oldest stages present.
- All larvae collected alive for rearing were dead on arrival at the laboratory as they were transported in sealed plastic pots inside air-tight evidence bags (Figure 1).
- Being asked to identify and determine the age of dried, flattened larvae 3.5 years post-collection, following their storage without preservative in a sealed glass jar kept in a fridge (remarkably, immersion in potassium hydroxide (KOH) [5] enabled them to be identified to species and assigned to a life stage) (Figure 2).

Lutz et al. [49] highlighted the importance of collecting insect evidence at the scene, especially the oldest stages, as in the second example above: in an analysis of 29 death investigations, they found that in 21 cases where puparia were collected at the scene, only in 14 (67%) of the subsequent autopsies were puparia collected. A published case that mirrors the last example given above is that of the re-examination of specimens in a "cold case" review nine years after the initial investigation [50]. Effective training of crime scene personnel with regular updating and use of recommended entomological collection equipment (e.g., [25]) are obvious ways to combat the illustrated complications of non-attendance of an entomologist at the scene.

## 3. Research and Casework—A Mutually Beneficial Relationship

Casework continuously identifies lacunae in our knowledge that research can address. Casework therefore poses questions and research can provide the solutions. Those solutions must be realistic, practical, validated and, ultimately, accepted in Court. However, researchers must not be limited at an early stage by what might seem impractical at the current time. For example, I sometimes questioned how practical for casework our own research using micro-computed tomography (micro-CT) to explore the intra-puparial development of the blow fly (Diptera: Calliphoridae) would be [51]. This research was stimulated by knowledge that the intra-puparial stages occupied at least 50% of the developmental period of blow flies, yet there were no adequate means available to estimate the stage reached by puparia collected from a scene. Micro-CT offers a means to virtually and non-destructively examine puparia, a bonus when this is forensic evidence, and can provide physiological aging at 10% intervals, equivalent to about one day at UK summer temperatures [52]. While a micro-CT is still an expensive capital item, it is increasingly possible to buy time on them, to use even remotely, and this trend is only likely to increase, as a survey of the use of CT in the field of forensic science reveals, showing a huge increase in publications in that area since 2000 [53]. Micro-CT in forensic entomology has great future potential and the research also develops our understanding of biology at a more fundamental level, e.g., of metamorphosis [54,55].

Examples of casework from my own experience that directly stimulated research include the following:

- Andrew Hart and I worked on two cases in quick succession in central and southern England in which the body of the victim was concealed inside a suitcase. In both cases there were fly larvae on the bodies and the question was, "can adult flies deposit their eggs on a suitcase in such a way that the larvae can develop on the body inside?" The subsequent research showed that not only can first instar larvae penetrate through suitcase zippers, but also that female flies can insert their ovipositors through the zippers and lay eggs inside the suitcase, enabling the hatching larvae to colonise the body [56].
- The oldest insect stages in a case in northern England in late Autumn were newly hatched first instar larvae from egg batches laid around a neck ligature and in the facial orifices. Most of the available information on blow fly egg stages gives a period from egg-laying to egg-hatching, therefore, aging of the specimens in this case was possible as they had just hatched: it was around six days due to the cool temperatures. However, if they had not hatched we would have struggled to age them and, at the low temperatures of this case or even lower, this embryonic period could be quite lengthy. Therefore, for use in similar future cases, we developed a simple method to estimate the age of *Calliphora vicina* (Diptera: Calliphoridae) eggs by morphological characters [57].
- An indoor case I attended in southern UK featured a large number of dead adult flies on the floor together with many dispersing larvae and puparia, but none of the latter in our collections were empty. Some adults were just emerging from puparia at the time of collection, so could we have overlooked empty puparia and was there any way of distinguishing if adults flies found at a scene had developed on the body or had, instead, flown in from outside? Developing the age-grading technique of wing fray, that was first used for tsetse flies (Diptera: Glossinidae) [58], we found that there were indeed major differences between the wing fray of populations of flies that emerged and died in a room, after developing as larvae on a body, compared with those that flew into the room from outdoors [59].

Much research is not stimulated by specific cases but arises from a general need recognized in all casework. For example, the provision of insect developmental data gathered in the laboratory and validated in the field [60], development of techniques for killing and preserving insect specimens in casework [61,62] and of methods for collecting temperature data at scenes of body recovery [63,64]. In most of these papers recommendations for practitioner protocols were included, such that in addition to the science there was guidance for those involved in casework.

New methods need to be validated before presentation in Court and peer review through scientific publication is one method of third-party examination of data, although not infallible. Validity of methods is a requirement highlighted in the Criminal Practice Directions for England and Wales which state, “Therefore factors which the court may take into account in determining the reliability of expert opinion, and especially of expert scientific opinion, include: (a) the extent and quality of the data on which the expert’s opinion is based, and the validity of the methods by which they were obtained;” [45] (19A.5, p. 33). With regard to new techniques, the Crown Prosecution Service of England and Wales sets out that, “Caution should always be exercised in assessing whether a new technique or novel science is accredited or is sufficiently sound to be admissible as evidence at trial”. One of the four factors to be considered in assessment was, “Whether the theory or technique has been subject to peer review and publication;” [13] (Part 1—Guidance: Admissibility of Expert Evidence, New or Novel Techniques).

At the time of writing, accreditation of individuals or organisations in England and Wales is only a requirement for DNA and fingerprint evidence [13] (Part 1—Guidance: Accreditation) [65]. Forensic science providers are assessed against ISO standards (especially ISO17020 and ISO17025) and the Codes of Practice and Conduct of the Forensic Science Regulator [44]. Some of the challenges around the accreditation of forensic entomology techniques and practitioners in the UK—e.g., validation of techniques, demonstration of practitioner competence and evaluating the strength of evidence—have been raised by Hall et al. [2]. However, it will surely only be a matter of time before some form of accreditation of individuals for casework in forensic entomology, and for their subsequent submission of evidence, is required, as appendices on entomology and other niche areas of forensic science have been drafted for the Forensic Science Regulator’s Code and will be considered in due course (G. Tully, personal communication). Indeed, the groundwork for this was laid in Europe with accreditation of the Department of Forensic Entomology of the Institut de Recherche Criminelle de la Gendarmerie Nationale, France, in 2007, the process for which is described by Gaudry and Dourel [66], focussing on preparation of documentation on good working standards and introduction of a quality assurance system. A thorough discussion of mandatory certification of forensic scientists in general from a United States perspective was provided by Melbourn et al. [67].

## 4. Challenges for the Future

Forensic science has faced a challenging last decade or more in the UK, due mainly to changes in the way forensic services are provided and reduced funding levels, prompting media headlines, such as “Forensics in Crisis” and “Police forensic science at ‘breaking point’”, e.g., [68,69]. Discussion of this dynamic situation has also been stimulated in the scientific literature, with commentaries that consider the impacts of closure of the UK Government’s Forensic Science Service in 2012 and the pressures that force forensic scientists in the commercial market to focus on chargeable casework at the expense of the broader aspects of science, including research and mentoring the next generation, e.g., [70,71].

To address the concerns highlighted above, a thorough review of the provision of forensic services in England and Wales was led by the Home Office, the Association of Police and Crime Commissioners and the National Police Chiefs’ Council [72]. One of its recommendations was to, “Ensure policing and the CJS [Criminal Justice System] benefits from advances in science and technology by developing and implementing new forensic techniques more coherently. Change is needed to bring about structured engagement across CJS partners, industry, science and academia in the testing, evaluation and development of new forensic techniques, improving the case for investment and helping forensic science providers to bring new innovation to market.” This essentially endorses the close links that should exist between research and casework.

The UK’s House of Lords conducted a similar review through its Science and Technology Select Committee [73]. Among its conclusions were recommendations to increase levels of funding for both technological advances and foundational research in forensic science and to create a National Institute for Forensic Science, to set priorities and oversee research on forensic science. One response to these recommendations was the formation of the Forensic Capability Network, designed and developed under the Transforming Forensic Programme [74], with the challenging yet exciting task of delivering high quality forensic science in England and Wales.

It is my opinion that there has never been a better time for more casework-related research, especially in areas which are not considered sufficiently "blue skies" to attract regular science funding but are essential "coal face" studies, for example, developmental studies, e.g., [75,76] of insect species that are frequently encountered in casework but are currently unavailable for use as evidence because of a lack of data. Areas for research could be provided by caseworkers in a valuable synergy, as exemplified by that reported in the social sciences some 70 years ago [77]. We also need to provide opportunities for young researchers to gain experience in forensic entomology casework and to receive appropriate recognition from academic employers of the value of casework. The relationship between research and casework seems clear, but the ability of personnel to move from the research end of the continuum to the casework end needs to be made more available and straightforward. One avenue that could be explored would be to copy the system for professional certification of forensic anthropologists adopted by the Royal Anthropological Institute, whereby aspiring, but inexperienced, forensic anthropologists are mentored by senior forensic anthropologists, enabling them to gain casework experience under expert tutelage [78]. Mentoring of students and early-career researchers by experienced forensic entomologists could improve the quality of entomology casework and, at an early stage, it could also be a highly influential factor in encouraging interest in the subject. This is demonstrated by the positive influence of mentors in stimulating the interest of medical students in a career in forensic pathology [79]. An article that discusses some of the challenges and rewards of a career as an expert witness in the forensic sciences concluded with a statement by Owen Jones, Professor of Law and Biological Sciences at the Vanderbilt Law School in Nashville, USA, saying, in effect, that legal systems will never be better informed than when those in science spend their time helping those systems advance along a more constructive and accurate path [80]. I agree strongly with that sentiment, but scientists, especially early-career researchers, need to be helped to do this in both their research and casework.

A useful and informative sheet summarising what an expert witness is describes an expert witness as one who, “may give opinion evidence within their expertise and in addition evidence of facts” [81]. Opinion evidence is clearly important, but the opinions expressed in an expert witness statement come from an interpretation of the facts of the case (e.g., the presence of larvae or the temperatures recorded) in the light of the expert witness’ experience and their knowledge of data (e.g., rates of insect development) that are generated through research focussed on supporting casework. This meld of experience and data is crucial so that the opinions are based on science rather than anecdote. Research and casework always work best together and the more the casework opinions can be based on and informed by research data, the better the value of the evidence delivered in court.

## Figures and Tables

**Table 1:** Some differences between research and casework in forensic entomology.

Areas of Difference	Research	Casework
Qualifications needed	PhD required	PhD and expert witness skills
Nature of study	Experimental replication	One-off, unique scenario
	Proactive	Reactive
	Planned schedule	Often highly disruptive
	Self-managed deadlines	Imposed, strict deadlines
Productions from study	Research publications	Expert witness statements
Rewards for production	Citation indices, academic credit	Often no academic credit but knowledge of the societal benefit

**Table 2:** Examples of differences between collection and analysis of forensic entomology evidence in an ideal world and in reality.

In an Ideal World the Forensic Entomologist:	In Reality There Are Cases Where:
visits the scene;	the forensic entomologist does not visit the scene;
collects a good range of insect evidence;	insect evidence collected is suboptimal in range;
preserves the insects appropriately;	insect evidence is not preserved properly;
retains some specimens alive for rearing;	specimens collected alive die before delivery;
collects scene meteorological data to compare with the nearest weather station;	no meteorological data is collected at the scene;
collects evidence at the start of the investigation;	evidence is only made available to the forensic entomologist months/years after the case;
prepares a robust expert witness statement.	it is extremely difficult to prepare a robust expert witness statement.
